# Characterization of Isoorientin and Paeoniflorin as Botanical Glucocorticoid Receptor Modulators from White Peony and Chasteberry

**DOI:** 10.3390/nu18101491

**Published:** 2026-05-07

**Authors:** Rasha M. Bashatwah, Luke T. Jesikiewicz, Alyssa L. Hardy, José A. Villegas, Kailiang Li, Brian T. Murphy, Joanna E. Burdette

**Affiliations:** 1 Department of Pharmaceutical Sciences, College of Pharmacy, University of Illinois Chicago, Chicago, IL 60612, USA; rbasha2@uic.edu (R.M.B.); ljesik@uic.edu (L.T.J.); ahardy20@uic.edu (A.L.H.); josev@uic.edu (J.A.V.); btmurphy@uic.edu (B.T.M.); 2 Department of Medicinal Chemistry and Pharmacognosy, Faculty of Pharmacy, Yarmouk University, Irbid 21163, Jordan

**Keywords:** botanical supplement, natural products, chasteberry, isoorientin, white peony, paeoniflorin, glucocorticoid receptor, progesterone receptor

## Abstract

Background/Objectives: Botanical supplements are increasingly investigated for their potential to address women’s health concerns. Compounds that modulate progesterone receptor (PR) signaling may help manage gynecologic disorders such as endometriosis, uterine hyperplasia, and preterm birth. Because PR ligands often cross-react with the glucocorticoid receptor (GR), this study examined two botanical compounds, paeoniflorin from *Paeonia lactiflora* (white peony root) and isoorientin from *Vitex agnus-castus* (chasteberry), that were identified as modulators of GR or PR signaling. Methods: Luciferase reporter assays were performed in OVCAR5, Ishikawa PR-B, and T47D A1-2 cells to evaluate GR and PR signaling. GR target gene expression was measured by qPCR. A receptor binding assay and computational docking were used to assess interaction with GR. Adipogenesis was evaluated in 3T3-L1 cells using Oil Red O staining and FABP4 protein expression by Western blot. Results: Paeoniflorin and isoorientin inhibited dexamethasone-induced GR signaling in OVCAR5 and Ishikawa PR-B cells. In T47D A1-2 cells, a variant of T47D engineered to express GR, both compounds blocked luciferase induction stimulated by progesterone; this effect was not observed in the parental line that expresses PR but lacks GR. In OVCAR5 cells, paeoniflorin or isoorientin combined with dexamethasone downregulated *GILZ* and *DUSP1/MKP1* mRNA. Isoorientin directly bound GR, and computational analysis supported potential binding poses. Both compounds also reduced lipid accumulation during 3T3-L1 adipocyte differentiation and decreased FABP4 expression, consistent with GR antagonist activity and reduced adipogenesis. Conclusions: These findings identify paeoniflorin and isoorientin as botanical modulators that suppress GR signaling and limit GR-dependent adipogenic responses across multiple cell-based models under controlled in vitro conditions.

## 1. Introduction

Therapies such as herbal remedies, botanical supplements, and products derived from natural sources often have significant biological implications for health. In a study measuring the prevalence and use of complementary and alternative medicinal approaches, the highest prevalence of roughly 65% was in the Obstetrics and Gynecology or “women’s health” field [1]. Women experience multiple symptoms associated with puberty, reproductive cycling, and in the postmenopausal setting that can be modified by botanicals, including premenstrual syndrome (PMS), endometriosis, polycystic ovarian syndrome (PCOS), dysmenorrhea, and infertility [2]. Botanical dietary supplements are often used due to their ease of administration, availability, good clinical efficacy, and affordability.

Progesterone is a steroid hormone secreted mainly from the gonads under the control of the pituitary–gonadal axis. It binds nuclear progesterone receptors (PR-A and PR-B) leading to receptor dimerization, nuclear internalization and interaction with progesterone response elements (PREs) to either activate or repress gene expression as part of the canonical pathway of this transcription factor [3]. Progesterone can also activate faster, non-genomic pathways to exert its activity through binding PR-C or membrane-bound receptors [3]. During the menstrual cycle, progesterone exerts pregestational effects by thickening the endometrial lining to support successful implantation. Progesterone deficiency can lead to increased myometrial contractility, higher miscarriage risk, preterm labor, decreased fertility [4] and endometriosis [5]. Moreover, the absence of progesterone during the menstrual cycle leads to unrestrained estrogen effects on the uterus, associated with a higher risk of endometrial hyperplasia and potentially endometrial cancer [6].

Glucocorticoids (GCs), primarily cortisol in humans, are steroid hormones produced by the adrenal glands that play a crucial role in regulating various physiological processes and metabolic adaptations, including stress response [7]. Glucocorticoids facilitate the early phases of preadipocyte differentiation by enhancing the adipogenic transcriptional cascade of CCAAT/enhancer-binding protein β and peroxisome proliferator-activated receptor gamma (C/EBPβ-PPARγ) induction, which subsequently leads to the induction of downstream adipocyte genes. Among these, fatty acid-binding protein 4 (FABP4/ap2) becomes highly expressed especially during terminal differentiation and in mature adipocytes, where it functions in fatty acid uptake, traffic into lipid droplets, and governing lipid accumulation and metabolic activity. Furthermore, elevated FABP4 levels can enter a feedback loop for PPARγ activity to limit additional differentiation to help stabilize the mature adipocyte pool under conditions of sustained lipid loading [8,9]. Increasing levels of circulating GCs have been implicated with female gynecological conditions such as PCOS [10] and stress-induced infertility [7] as well as metabolic syndrome conditions like dyslipidemia [11]. Managing the levels of GCs in the body either by decreasing the level of circulating GCs or modulating the GR can prove beneficial in these conditions.

Hormone receptors exhibit complex crosstalk through multiple mechanisms at both the protein–protein interaction and chromatin-binding levels [12]. Progesterone, androgen, glucocorticoid and mineralocorticoid receptors share remarkable structural similarity, with approximately 55% sequence identity in their ligand-binding domain [13]. Moreover, these four receptors recognize nearly identical consensus DNA sequences; they all bind the same hormone response elements (HREs). Despite their architectural similarity, these receptors exhibit distinct binding patterns leading to distinct downstream physiological effects. PR and GR can bind an overlapping set of chromatin binding regions and recruit shared chaperones and cofactors, implying a cooperative or competitive behavior when multiple receptors are activated in the same cell [14]. For example, in breast cancer, Ogara et al. have found that GR antagonizes PR activity through ligand-dependent mechanisms: R5020-bound GR redistributes to a limited set of shared binding sites and forms GR-PR complexes at select enhancers, whereas Dex-bound GR occupies numerous HREs shared with PR, increasing GR-PR interaction and collectively suppressing PR-mediated transcription and proliferation [12]. Similarly, in the uterus, GR and PR exhibit opposing interaction; Austin et al. reported that baicalein from the roots of skullcap (*Scutellaria baicalensis)* functions as a PR antagonist with simultaneous GR agonist activity [15]. Irilone from red clover (*Trifolium pratense* L.) was shown to have a tissue-dependent PR-potentiating effect, acting through the estrogen receptor (ER) in breast cells and through GR in endometrial cells [2]. This multi-target activity underlies the therapeutic potential of botanical use in reproductive health applications. While many botanicals exhibit polypharmacological effects on hormone receptors, they also often exhibit tissue-dependent and concentration-dependent effects.

White peony (*Paeonia lactiflora* Pall.), also known as Bai Shao in Traditional Chinese Medicine (TCM), has been used for over two millennia in gynecological practice, primarily for its effects on hormone-responsive health conditions. Several formulations containing white peony roots are used to address menstrual irregularities, dysmenorrhea, and endometriosis [16,17,18]. Paeoniflorin (PFL), a monoterpene glucoside, is the most abundant biologically active metabolite in white peony and a marker for its identification, and has been linked to the amelioration of PCOS symptoms in an in vivo rat model where PCOS was Dehydroepiandrosterone-induced [19]. PFL has also been shown to reduce Dex-induced testosterone levels in primary murine theca cells by affecting cytochrome P450-17A1 expression levels [20]. Moreover, PFL was found to enhance the hypothalamic–pituitary–adrenal negative feedback mechanism, increasing the levels of GR in the amygdala while decreasing it in the hippocampus, indicating tissue-specific activity of PFL in the body [21].

Chasteberry extract (*Vitex agnus-castus* L.) has also been used for managing premenstrual syndrome, PCOS, and endometriosis [22,23]. Chasteberry extracts have been shown to inhibit prolactin release in vitro, a mechanism attributed to their dopamine agonist effect on the dopamine D_2_ receptor [24]. Moreover, chasteberry extracts have been shown to indirectly stimulate the secretion of luteinizing hormone (LH), leading to an increase in progesterone and 17β-estradiol [25]. Chasteberry extracts have also been reported to contain estrogen-mimicking compounds [26,27]. Fukahori et al. established an HPLC fingerprint for chasteberry extracts, which showed 26 characteristic peaks, and isoorientin, a C-glucosyl flavone, was identified as a pharmacopeial marker [28]. The parent aglycone of isoorientin, luteolin, exhibits potent estrogenic activity, moderate anti-progestogenic activity, and weak anti-glucocorticoid activity [29].

This study characterizes the effects of paeoniflorin and isoorientin on GR and PR signaling in vitro using an HRE-luciferase (HRE-Luc) assay across multiple cell lines and downstream RT-qPCR validation, as well as a functional metabolic assay in 3T3-L1 cell line to determine whether these botanical compounds modulate lipid accumulation in differentiated adipocytes. Overall, we identified isoorientin as a GR antagonist in multiple cell lines based on both receptor binding and computational modeling.

## 2. Materials and Methods

### 2.1. Small Molecules and Reagents

Dexamethasone (11015), RU486 (10006317), isoorientin (26862), and IBMX (13347) were purchased from Cayman Chemical, Ann Arbor, MI, USA. Progesterone (P0130), paeoniflorin (P0038), and dimethyl sulfoxide (DMSO) (D8418) were purchased from Sigma Aldrich (St. Louis, MO, USA). All small molecules were dissolved in DMSO at a 1000× concentration to make a stock solution, ensuring a maximum of 0.1% DMSO upon treatment. For combination treatments, higher concentrations of stock solutions were prepared and used. The concentrations used throughout our work mirror doses that robustly activate steroid receptors, as determined by our assay optimization.

### 2.2. Cell Culture

OVCAR5 cells (American Type Culture Collection, ATCC, Manassas, VA, USA) were maintained in RPMI 1640 (10-040-CV, Corning, Corning, NY, USA) supplemented with 10% FBS, 2 mM L-glutamine, and penicillin/streptomycin (100 U/100 µg/mL, cat# 15070063, ThermoFisher, Waltham, MA, USA). Ishikawa cells stably expressing PR-B under a CMV promoter were maintained in phenol red-free DMEM/F12 media (11039–021, Invitrogen, Waltham, MA, USA) and supplemented with 5% charcoal–dextran double-stripped FBS and selected by 250 μg/mL of G418 (A1720, Sigma Aldrich, St. Louis, MO, USA) and 125 μg/mL hygromycin B (H3274, Sigma Aldrich, St. Louis, MO, USA). Ishikawa PR-B cells were generously donated by Dr. Leen Blok, Department of Obstetrics and Gynecology, Erasmus, Medical Center, Rotterdam, The Netherlands [30]. T47D cells (ATCC, Manassas, VA, USA) were maintained in RPMI 1640 (10-040-CV, Corning, Corning, NY, USA) supplemented with 10% FBS, 2 mM L-glutamine (25030081, Thermo Scientific, Waltham, MA, USA), and penicillin/streptomycin (100 U/100 µg/mL). 3T3-L1 murine preadipocyte cells (ATCC, Manassas, VA, USA) were maintained in DMEM media supplemented with 10% calf serum and penicillin/streptomycin (100 U/100 µg/mL). All cells were maintained at 37 °C in a humidified incubator and 5% CO_2_.

T47D and OVCAR5 cells were maintained in steroid-free media, which lacked phenol red and had 5% dextran-coated charcoal-treated FBS, 24 h before starting the experiments involving hormone treatments.

### 2.3. Luciferase Reporter Assay

Cells were plated in phenol red-free, charcoal–dextran-stripped FBS media in 24-well plates (Corning, Corning, NY, USA) and left to recover overnight. The next day, cells were transfected with a mixture of HRE-luciferase plasmid and RSV-β-galactosidase at a ratio of 2:1 using TransIT LT1 transfection reagent (Mirus Bio, Madison, WI, USA) overnight according to the manufacturer’s protocol. Cells were then treated for 24 h with the hormones as a positive control, DMSO as the vehicle control, and the botanicals at various concentrations. Post-treatment, the cells were lysed (2% Triton-X, 0.1% DTT in GME buffer) and frozen for an hour, and then the lysate was divided into two plates for the luciferase and β-galactosidase assays as previously described [15]. Luciferase readings were normalized to the β-galactosidase activity then to the vehicle control and reported as mean fold change ± SEM of at least three biological replicates.

### 2.4. Quantitative PCR

OVCAR5 cells were seeded at 200,000 cells/well in a 6-well plate in phenol red-free and stripped media for 24 h (~70% confluency). The cells were treated with paeoniflorin and isoorientin (20 µM) for 30 min, followed by the addition of Dex (30 nM) for 6 h. RNA was extracted using TRIzol (Life Technologies, Grand Island, NY, USA) and chloroform with isopropanol precipitation, followed by ethanol washes. The RNA was reverse transcribed to cDNA using an iScript™ cDNA synthesis kit (1708891, Bio-Rad, Hercules, CA, USA) following the manufacturer’s protocol. Quantitative PCR was performed using PowerUp™ SYBR™ Green Master Mix for qPCR (1725271, Bio-Rad, Hercules, CA, USA) according to the manufacturer’s protocol on the CFX connect Real-Time PCR Detection System (Bio-Rad, Hercules, CA, USA). Transcript expression was calculated using the ΔΔCt method and samples were normalized to the reference gene glyceraldehyde-3-phosphate dehydrogenase (*GAPDH*). Treatment readings were normalized to the vehicle control (DMSO), set to 1, and results were reported as mean fold change ± SEM from at least 3 biological replicates. *GILZ* and *MKP1/DUSP1* primers (Supplementary Materials Table S1) were designed using the NCBI Primer-Blast tool (https://www.ncbi.nlm.nih.gov/tools/primer-blast/ accessed on 16 September 2024, version 2.5.0) and ordered from Integrated DNA technologies (IDT, San Diego, CA, USA).

### 2.5. Western Blot

Cells were seeded and allowed to adhere overnight in phenol red-free media supplemented with charcoal-stripped FBS. The cells were treated for 24 h then lysed with a lysis buffer containing RIPA, a protease inhibitor and a phosphatase inhibitor. Protein concentration was assessed via the Bradford protein assay, and the samples (30 µg of protein) were run on SDS-PAGE gels by electrophoresis, then transferred to a nitrocellulose membrane. The membrane was blocked in non-fat milk followed by primary antibody incubation at 4 °C (Supplementary Materials Table S2). After 24 h, the membranes were washed and incubated with the appropriate secondary antibody and developed using SuperSignal^®^ West Femto Maximum sensitivity substrate (PI34096, ThermoFisher, Waltham, MA, USA) on the Azure 300 Chemiluminescent Western Blot Imager (Azure Biosystems Inc., Dublin, CA, USA) [15]. The images were quantified using ImageJ (NIH) and plotted using GraphPad Prism (Version 10.6.0 for Windows, GraphPad Software, Boston, MA, USA) after normalization. Data is presented as an average of 3 biological replicates ± SEM, and statistical analysis was done using one-way ANOVA followed by Tukey’s post hoc test, where the *p*-value was set to be 0.05.

### 2.6. 3T3-L1 Cell Differentiation into Adipocytes

To initiate cell differentiation, the media was switched to DMEM supplemented with 10% FBS and penicillin/streptomycin (100 U/100 µg/mL). 3T3-L1 cells were seeded and allowed to reach 100% confluence, which marked day zero (D0). At D0 the cells were incubated with the differentiation media (DM) containing 1 µM Dex, 1 µg/mL insulin, and 0.5 mM IBMX, as previously described [31], for 3 days. Following that, the cells were incubated in maintenance media (1 µg/mL insulin) for 2 days. Afterwards, the media was changed to DMEM supplemented with FBS and pen/strep until the experiment was stopped at D10. Treatments were added to the cells starting from D0.

### 2.7. Oil Red O Assay for Lipid Accumulation (ORO)

3T3-L1 cells were differentiated according to the protocol mentioned above for 10 days then they were washed and fixed with 4%. The cells were then washed and dehydrated with 60% isopropanol and stained with 60% ORO for 20 min. The dye was initially dissolved in 100% isopropanol then diluted to 60% in distilled water. The wells were then washed until the stain was cleared and allowed to air-dry. For quantification, the stain was redissolved in 300 µL isopropanol at RT; then, absorbance was measured at 500 nm. The data shown represents the mean ± SEM of at least three biological replicates. The images of the wells are taken by a Nikon Eclipse TE200 (Tokyo, Japan) via an AmScope microscope digital camera (Irvine, CA, USA).

### 2.8. Radioligand GR Binding Assay

Affinity of paeoniflorin and isoorientin for the human GR was evaluated in a GR (h) agonist radioligand receptor binding assay performed by Eurofins Cerep (Celle l’Evescault, France; Study No. 100078167, Study ID US034-0028622) according to their protocol as described by Clark et al. [32]. Human GR was assessed in cytosolic preparations from IM-9 human B lymphoblast cells endogenously expressing GR, incubated with [^3^H] Dex (at a final concentration of 1.5 nM, Kd 1.5 nM) for 24 h at 4 °C in the presence of the test compounds or vehicle (DMSO), with triamcinolone (10 µM) used to define non-specific binding. The bound radioligand was quantified by scintillation counting. Specific binding in the presence of paeoniflorin and isoorientin was quantified relative to control samples and used to generate a concentration–response curve for the IC_50_ and K_i_ determination.

The concentration–response data for paeoniflorin and isoorientin were analyzed by non-linear regression using Cerep’s Hill software (Eurofins Discovery, Celle L’Evescault, France), validated by comparison with SigmaPlot^®^ 4.0 for Windows^®^ (SPSS Inc., Chicago, IL, USA), to determine IC_50_ values. Inhibition constants (K_i_) were calculated from IC_50_ values using the Cheng–Prusoff equation, with ligand concentration and K_d_ as specified above. The results were plotted using GraphPad Prism and the points represent the average of two biological replicates.

### 2.9. Docking and Classical Molecular Dynamics

The structure of isoorientin was optimized using Gaussian09 [33] with the B3LYP functional and a 6-31G(d) basis set. The structure of the antagonist form of the GR was taken from the PDB (access code 3H52) [34]. Missing residues were added to the structure of chain A using the ModLoop server [35]. The protonation state of charged residues was determined using the H++ webserver (version 4.0) at pH 7 and 0.15 μmol salinity [36]. Each small molecule was docked in the GR using the Autodock suite (version 4.2.6) [37] and the Lamarckian genetic algorithm. The docking parameters were genetic algorithm run of 30, population size of 150, and 25 million energy evaluations.

Classical molecular dynamics (MD) simulations were carried out using the pmemd module of the GPU-accelerated Amber22 package [38]. The Amber ff14SB force field [39] was used for standard residues; TIP3P was used for solvent water molecules and ions. We used the Merz–Singh–Kollman scheme for RESP charge fitting of electrostatic potential generated at the B3LYP/6-31G(d) level of theory for each ligand, utilizing the generalized Amber force field (gaff) [40] to generate forcefield parameters for the substrate. The ligand–receptor complex was solvated in a square water box with a periodic boundary condition with the minimum distance between the peptide assembly and the edge of the box as 12 Å. The classical MD simulations follow four stages; minimization, heating, equilibration, and production runs. We applied 30,000 steps of energy minimization with 5.0 kcal mol^−1^ Å^−2^ restraints on the receptor and ligand. Then, the system was heated to 300 K over 10,000 steps of MD at a 1fs timstep with the same 5.0 kcal mol^−1^ Å^−2^ restraints. The system was then equilibrated over 300,000 steps of MD at a 1fs timestep, gradually releasing the harmonic restraints every 50,000 steps.

Production simulations were performed at constant pressure with the Beresden barostat and 300 K (Lagevin thermostat) with a 2 fs timestep with 0.1 kcal mol^−1^ Å^−2^ restraints on the backbone atoms of the GR only. Production runs were performed for 100 ns in triplicate, employing the SHAKE algorithm for H atoms, the Particle-Mesh Ewald method [41] for long-range electrostatic effects, and an 8.0 Å cutoff for electrostatic interactions. Frames were written to the file every 5000 steps (10,000 frames per simulation).

### 2.10. Molecular Dynamics Analysis

CPPTRAJ (version 6.18.1) [42] was utilized for RMSD calculations as well as interaction distances and frequencies. Clustering analysis based on the root-mean-square deviation (RMSD) of the receptor backbone was carried out using the CPPTRAJ module to identify the most populated ligand conformation in the MD simulations of all three replicas. H-bonds are specified as having a donor–acceptor atom distance of <3.0 Å and a bond angle of >140°. H-bond frequency is reported as the number of frames specifying H-bond criteria across the three triplicate runs of MD simulation divided by the total number of simulation frames. 3D renderings were created using PyMOL (version 3.0.0) [43].

### 2.11. Binding Energy Calculations

Binding energy calculations were computed using the MPI implementation of the mmgbsa.py [44] module (version 14.0) in AmberTools22 with igb = 8 and surface tension set to 0.0072. Energetic sampling for MMGBSA was for 0.2 ns in simulation within a representative 30 ns segment. Normal mode entropy calculations were performed on 15 frames taken every 2 ns within the 30 ns simulation windows. This was done for all reported energy values except for isoorientin pose 1 (state 1 and 2), where 80 ns of simulation was used with nmode sampling every 8 ns (30 frames for nmode).

## 3. Results

### 3.1. Paeoniflorin and Isoorientin Inhibit GR HRE-Luc Activity in GR-Expressing Cells

Both paeoniflorin and isoorientin serve as chemical markers to identify extracts from *Paeoniaceae* and chasteberry, respectively. Prior to functional testing, lysates from OVCAR5, T47D, T47D A1-2, and Ishikawa PR-B cells were analyzed by Western blot to confirm receptor expression. OVCAR5, Ishikawa PR-B and T47D A1-2 all express GR [15] (Figure S1), whereas T47D cells express both PR isoforms (PR-A and PR-B), ER and no GR [12]. Ishikawa PR-B stably expresses PR-B under CMV control developed by the Blok Lab [30].

**Figure 1:**
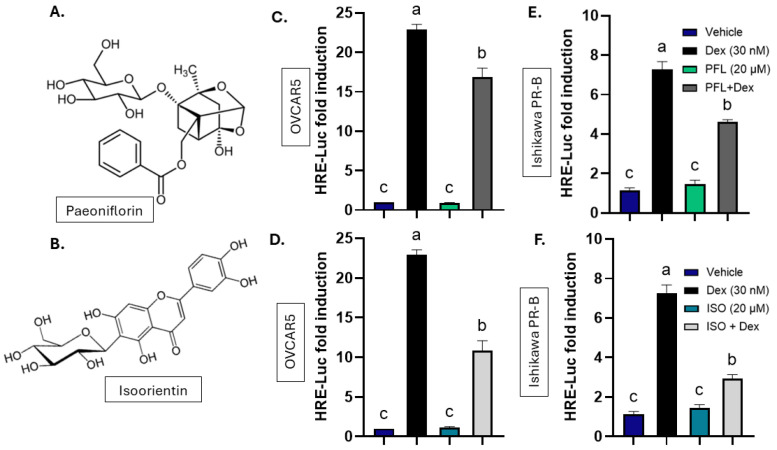
Paeoniflorin and isoorientin inhibit GR in OVCAR5 and Ishikawa PR-B. (**A**) Structure of paeoniflorin (PFL). (**B**) Structure of isoorientin (ISO). (**C**,**D**) Hormone response element-luciferase (HRE-Luc) assay in OVCAR5 cells treated with vehicle (0.1% DMSO), 30 nM Dexamethasone (Dex) and (**C**) 20 µM PFL alone or in the presence of 30 nM or (**D**) 20 µM ISO alone or in the presence of 30 nM Dex. (**E**,**F**) HRE-Luc assay in Ishikawa PR-B cells treated with vehicle, 30 nM Dex, and (**E**) 20 µM PFL alone or in the presence of 30 nM or (**F**) 20 µM ISO alone or in the presence of 30 nM Dex. All treatments last 24 h. N ≥ 3. Data represent mean ± SEM. Letters indicate results of one-way ANOVA with Tukey’s post hoc analysis, *p* < 0.05.

The HRE-Luc reporter assay was then used to define the impact of paeoniflorin and isoorientin (structure shown in Figure 1A and 1B respectively) on GR-mediated signaling. Dex (30 nM) was used as a positive control to induce GR activity in OVCAR5 and it induced the HRE-Luc assay 22.9-fold (*p* < 0.0001, Figure 1C). Paeoniflorin alone did not activate the reporter, indicating the absence of GR agonist activity, whereas co-treatment with paeoniflorin (20 µM) and Dex reduced Dex-induced luciferase activity by 26.5% (*p* < 0.005, Figure 1C). Similarly, isoorientin alone did not activate the reporter, but co-treatment with isoorientin (20 µM) and Dex decreased the GR-driven HRE-Luc activity by 52.5% (*p* < 0.0001, Figure 1D), demonstrating that both botanicals attenuate Dex-induced GR signaling in OVCAR5 cells.

To examine GR signaling in a cell line that also expresses PR, Ishikawa PR-B cells were transfected with the same HRE-Luc construct. Paeoniflorin again failed to activate the reporter, consistent with a lack of agonist activity of GR or PR in this assay. Treatment with 30 nM Dex produced a 6.9-fold induction of luciferase activity (*p* < 0.0001, Figure 1E), and co-treatment with 20 µM paeoniflorin reduced the response by 33.5% (*p* = 0.0108, Figure 1E). Isoorientin at 20 µM did not activate the reporter on its own in Ishikawa PR-B, indicating it does not activate either GR or PR, but a co-treatment with 20 µM isoorientin plus 30 nM Dex inhibited the Dex-induced signal by 58% (*p* < 0.0001, Figure 1F). To determine whether these inhibitory effects were dose-dependent, additional concentrations of paeoniflorin and isoorientin were tested in combination with Dex. Lower and higher concentrations of paeoniflorin did not block Dex activity significantly, whereas 10 µM isoorientin already produced measurable inhibition of HRE-Luc activity in OVCAR5 cells (Figure S2).

### 3.2. Paeoniflorin and Isoorientin Inhibit PR in Breast Cancer Cell Line Expressing PR and GR

Ligand-bound GR can interact functionally with PR, and this crosstalk is dependent on tissue-specific receptor expression patterns, stromal context, and differential regulator recruitment and activity. To evaluate the effects of paeoniflorin and isoorientin on PR signaling in a GR-positive background, two PR/GR co-expressing models were used: the endometrial cell line Ishikawa PR-B and the breast cancer cell line T47D A1-2. Both cell lines were transfected with the HRE-Luc reporter and treated with progesterone (P_4_) as a positive control for PR-dependent transcriptional activity.

**Figure 2:**
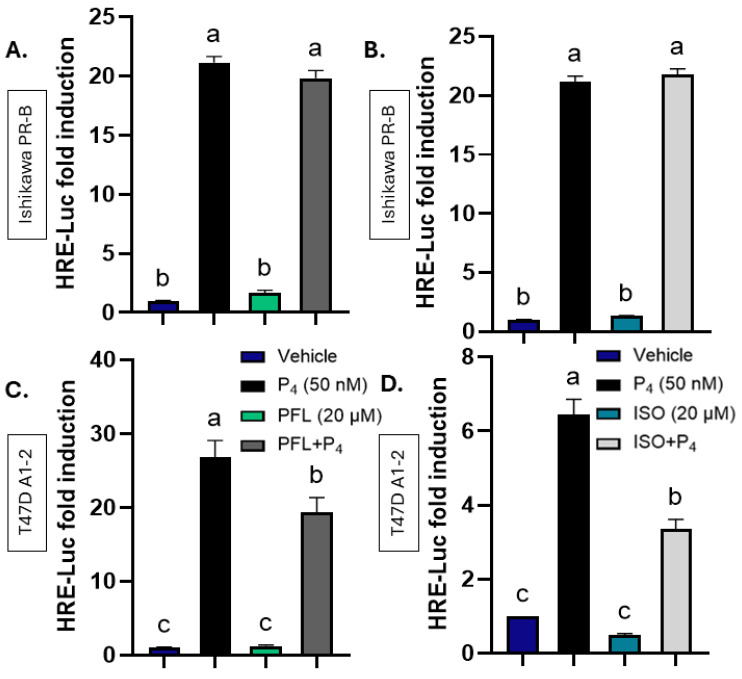
Paeoniflorin and isoorientin block progesterone receptor (PR) activity in T47D A1-2 expressing GR, but not in Ishikawa PR-B. (**A**,**B**) HRE-Luc assay in Ishikawa PR-B cells treated with vehicle (0.1% DMSO), 50 nM progesterone (P_4_) and (**A**) 20 µM PFL in the presence of 50 nM P_4_; (**B**) 10 µM ISO in the presence of 50 nM P_4_. (**C**,**D**) HRE-Luc assay in T47D A1-2 cells treated with vehicle, 50 nM P_4_ and (**C**) 20 µM PFL alone or 20 µM PFL in the presence of 50 nM P_4_ or (**D**) 10 µM ISO alone and 10 µM ISO in the presence of 50 nM P_4_. All treatments last 24 h. PN ≥ 3. Data represent mean ± SEM. Letters indicate results of one-way ANOVA with Tukey’s post hoc analysis at *p* < 0.05.

In Ishikawa PR-B cells, treatment with 50 nM P_4_ induced an average 21-fold increase in luciferase activity (*p* < 0.0001, Figure 2A), and co-treatment with 20 µM of paeoniflorin with P_4_ did not alter this response (Figure 2A). Likewise, co-treatment with 20 µM of isoorientin and P_4_ had no inhibitory effects on the P_4_-induced luciferase signal (Figure 2B), indicating that neither compound measurably inhibits P_4_-driven activity through PR in Ishikawa PR-B.

In contrast, a different pattern emerged in T47D A1-2 cells that have been engineered to express GR. The ligand P_4_ alone induced the reporter 30.8-fold (*p* < 0.0001, Figure 2C). Paeoniflorin (20 µM) alone did not affect basal luciferase levels, but a combination of 20 µM paeoniflorin and 50 nM P_4_ led to an inhibition of the luciferase signal by 37.3% (*p* < 0.0017, Figure 2C). Isoorientin alone at 20 µM also failed to activate the reporter by itself, yet co-treatment with 20 µM isoorientin and 50 nM P_4_ inhibited the P_4_ response by 47.7% (*p* < 0.0001, Figure 2D). These data suggest that GR-bound botanicals could block PR signaling.

### 3.3. Paeoniflorin and Isoorientin Do Not Modulate the Estrogen Receptor

**Figure 3:**
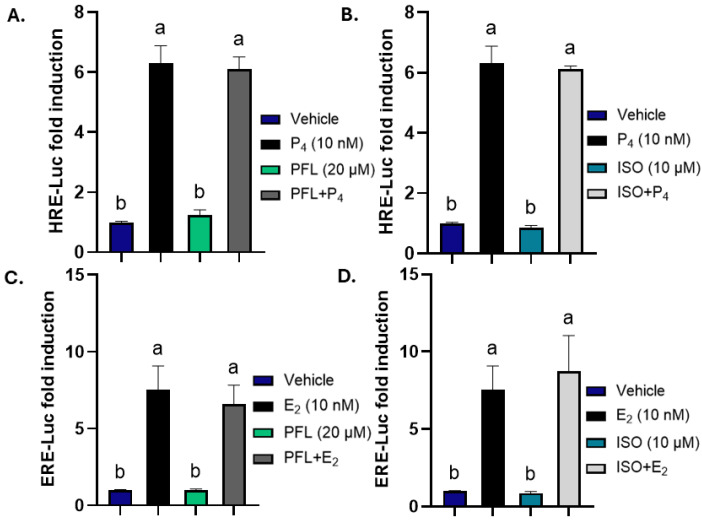
Paeoniflorin and isoorientin do not modulate estrogen (ER) or progesterone (PR) receptor activity in T47D cell line. (**A**,**B**) HRE-Luc assay in cells treated with vehicle (0.1% DMSO), 10 nM P_4_ and (**A**) 20 µM PFL alone or in the presence of 10 nM P_4_; (**B**) 10 µM ISO alone or in the presence of 10 nM P_4_. (**C**,**D**) Estrogen response element (ERE)-Luciferase assay in cells treated with vehicle, 10 nM Estradiol (E_2_) and (**C**) 20 µM PFL alone or in the presence of 10 nM E_2_; (**D**) 10 µM ISO alone or in the presence of 10 nM E_2_. All treatments last 24 h. P_4_, E_2_, PFL and ISO are dissolved in DMSO at a final concentration of 0.1%. N ≥ 3. Data represents mean ± SEM. One-way ANOVA with Tukey’s post hoc analysis; groups not sharing a letter differ at *p* < 0.05.

The cell line T47D A1-2 also expresses ER, raising the possibility that the inhibitory effects on P_4_-driven luciferase activity might involve ER rather than GR-PR crosstalk. To address this, the HRE-Luc assay was performed in the parental T47D line. Treatment with 10 nM of P_4_ used as positive control induced a 6.3-fold increase in luciferase activity (*p* < 0.0001, Figure 3A). Paeoniflorin alone did not induce luciferase activity at 20 µM and co-treatment with 10 nM P_4_ did not inhibit the P_4_ response (Figure 3A). Similarly, 10 µM isoorientin neither activated the reporter nor reduced P_4_-induced activity when combined (Figure 3B), indicating that the inhibitory effects observed in T47D A1-2 require GR and are not mediated by ER in the parental T47D cell line.

To further evaluate direct ER modulation, T47D cells were transfected with an estrogen response element-driven luciferase construct (ERE-Luc). Cells were treated with the vehicle, 10 nM estradiol (E_2_) as a positive control, or E_2_ in combination with the botanicals. Estradiol induced a 7.5-fold increase in luciferase activity (*p* < 0.0001, Figure 3C). Paeoniflorin (20 µM) alone did not alter ER activity; when in combination with E_2_, it also did not alter E_2_-induced activity (Figure 3C), and isoorientin (10 µM) likewise failed to activate ER or inhibit E_2_-driven luciferase activity (Figure 3D).

### 3.4. Paeoniflorin and Isoorientin Downregulate a Subset of GR-Controlled Genetic Transcripts

**Figure 4:**
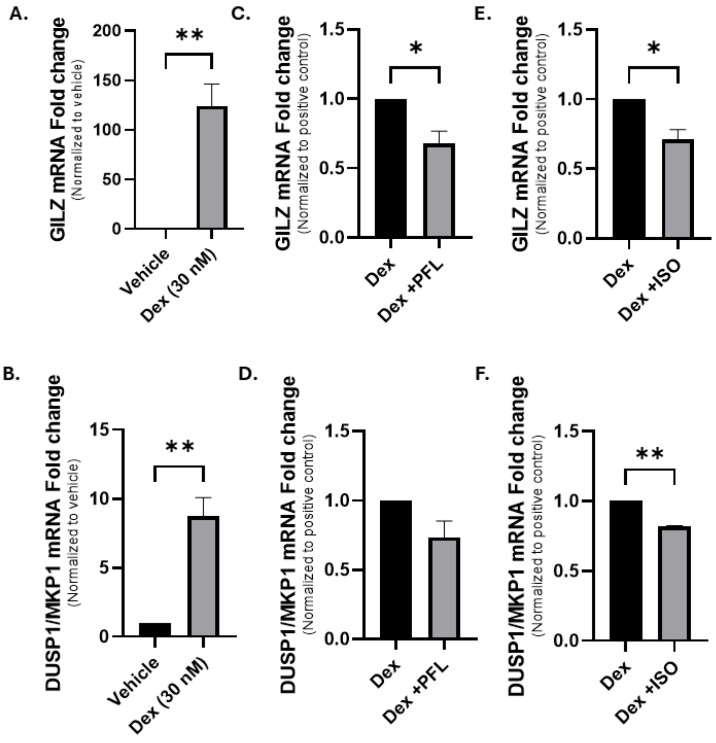
Paeoniflorin and isoorientin downregulate GR-regulated transcripts in OVCAR5 cells via qPCR. (**A**) *GILZ* and (**B**) *DUSP1/MKP1* in cells treated with 30 nM Dex for 6 h, where the control was the vehicle-treated cells (0.1% DMSO). (**C**,**D**) Fold change in levels of (**C**) *GILZ* and (**D**) *DUSP1/MKP1* in cells treated with a combination of 30 nM Dex and 20 µM PFL. Fold change in levels of (**E**) *GILZ* and (**F**) *DUSP1/MKP1* in cells treated with a combination of 30 nM Dex and 20 µM ISO. N ≥ 3. The values represent the mean ± SEM. Significant values were determined by a Student *t*-test; * *p* < 0.05, ** *p* < 0.01 relative to control.

GR is a ligand-activated transcription factor that controls a subset of genes. Glucocorticoid-induced leucine zipper (*GILZ*) and dual-specificity phosphatase 1 gene encoding for mitogen-activated protein kinase phosphatase 1 (*DUSP1/MKP1*) are well-characterized genes regulated by GR and induced by Dex [45,46]. To evaluate whether paeoniflorin or isoorientin block GR transcriptional output, RT-qPCR was performed in OVCAR5 cells. Treatment with 30 nM Dex for 6 h increased *GILZ* mRNA by an average of 124-fold and *DUSP1/MKP1* by 8.7-fold relative to vehicle control (0.1% DMSO) (Figure 4A and 4B, respectively). A co-treatment of Dex with 1 µM RU486 led to a decrease of 96% in *GILZ* and 63% in *DUSP1/MKP1* mRNA abundance (Figure S3). Co-treatment with 20 µM paeoniflorin and Dex attenuated these responses. *GILZ* expression was reduced by 37%, corresponding to a mean 0.67-fold change when normalized to Dex-treated cells (Figure 4C), while *DUSP1/MKP1* levels decreased by 26.5%, corresponding to a 0.74-fold change (Figure 4D). Similarly, co-treatment with 20 µM isoorientin and Dex decreased *GILZ* expression by 29% (0.71-fold relative to Dex, Figure 4E) and *DUSP1/MKP1* by 15% (0.85-fold relative to Dex, Figure 4F). These data indicate that both paeoniflorin and isoorientin partially antagonize Dex-induced GR target gene activation in OVCAR5 cells.

### 3.5. Paeoniflorin and Isoorientin Decrease Lipid Accumulation in 3T3-L1 Adipocytes

**Figure 5:**
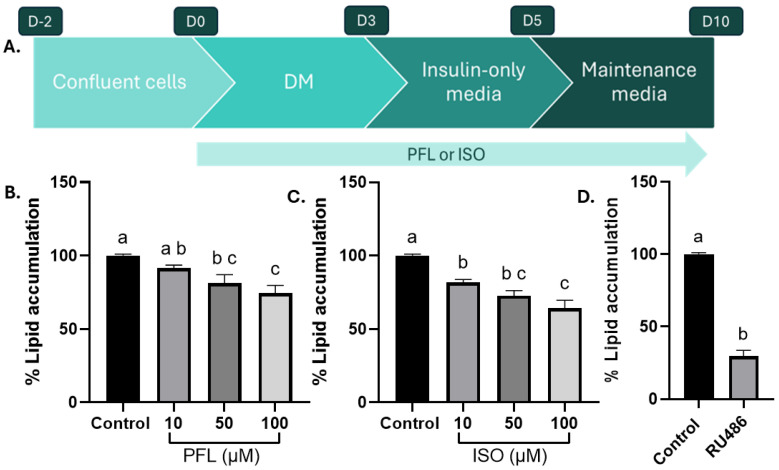
Paeoniflorin and isoorientin decrease lipid accumulation in 3T3-L1 differentiated adipocytes by attenuating dexamethasone-driven GR signaling. (**A**) Experimental design for differentiating 3T3-L1 cells. Cells were seeded and left to reach 100% confluence for two days, then differentiated using a mix of IBMX (0.5 mM), Dex (1 µM), and insulin (1 µg/mL) for 3 days, and switched to maintenance media containing insulin (1 µg/mL) for 2 days, followed by regular media for 5 days. The treatments with PFL, ISO or RU486 spanned over the whole differentiation period. (**B**–**D**) Oil-Red O quantification of differentiated adipocytes being treated with (**B**) PFL or (**C**) ISO or (**D**) RU486 (10 µM). Oil-Red O assay measured at 500 nm from stained lipids accumulated in the adipocytes. N ≥ 3. Data represents mean ± SEM. One-way ANOVA with Tukey’s post hoc analysis, groups not sharing a letter differ at *p* < 0.05.

Glucocorticoid inhibition in adipocytes reduces lipid accumulation during differentiation. To determine whether paeoniflorin or isoorientin can block GR activity in differentiating adipocytes and thereby limit lipid deposition, 3T3-L1 cells were induced to differentiate using the experimental design shown in Figure 5A. Intracellular lipid accumulation was quantified by Oil Red O (ORO) staining followed by dye elution. Compared to differentiated control cells, both compounds produced a clear, concentration-dependent reduction in ORO signal, indicating decreased neutral lipid deposition within the lipid droplets. The treatment with paeoniflorin showed a decrease in lipid content to approximately 92%, 81% and 74%, respectively (*p* < 0.05 for 50 µM and <0.01 for 100 µM, Figure 5B). Similarly, isoorientin lowered lipid accumulation levels to 82%, 73% and 64%, respectively (*p* < 0.01 for 50 µM and *p* < 0.001 for 100 µM, Figure 5C). Notably, a treatment with 10 µM RU486 mimicked this reduction in Oil-Red O staining to 30% compared to differentiated control (*p* < 0.001, Figure 5D), supporting the notion that inhibition of GR signaling contributes to the anti-adipogenic effects of paeoniflorin and isoorientin in 3T3-L1 adipocytes. Moreover, differentiating 3T3-L1 adipocytes treated with paeoniflorin or isoorientin appeared smaller and contained fewer visible and measurable lipid droplets than the control differentiated cells (Figure S4). Previous studies have shown that neither PFL nor ISO is toxic to 3T3-L1 cells [47,48].

### 3.6. Isoorientin Downregulates Levels of FABP4, a Fatty Acid-Binding Protein in 3T3-L1 Cells

**Figure 6:**
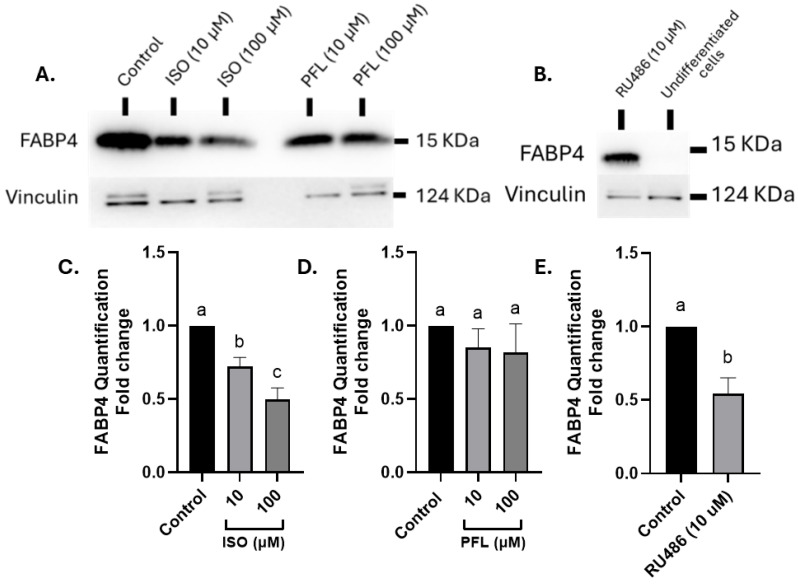
Isoorientin downregulates levels of FABP4 in 3T3-L1 cells. (**A**,**B**) Representative Western blot for FABP4 levels in 3T3-L1 cells. (**A**) A Western blot of the control (differentiated cells), cells treated with ISO at 10 and 100 µM, and cells treated with PFL at 10 and 100 µM. Vinculin was used as a loading control. (**B**) A Western blot of cells treated with RU486 (a GR antagonist) at 10 µM, and undifferentiated cells. (**C**–**E**) Densitometry of the Western blot images showing the levels of FABP4 in cells treated with (**C**) 10 and 100 µM ISO, (**D**) 10 or 100 µM PFL and (**E**) 10 µM RU486. The membranes were cut based on molecular weight markers and incubated separately with the primary antibodies. N ≥ 3. Data represents mean ± SEM. One-way ANOVA with Tukey’s post hoc analysis for panels (**C**,**D**) and unpaired *t*-test for panel (**E**). Groups not sharing a letter differ at *p* < 0.05.

FABP4 expression steadily increases during adipogenesis, helping to support lipid uptake and fat droplet growth, and blocking GR disrupts this sequence by disrupting the expression of FABP4 in the cells. To determine whether this phenotype reflected reduced FABP4-associated lipid accumulation, FABP4 protein abundance was assessed by Western blot in cells differentiated in the presence of 10 µM and 100 µM of paeoniflorin, 10 µM and 100 µM of isoorientin (Figure 6A), or 10 µM of RU486 (Figure 6B). Isoorientin significantly decreased FABP4 protein in a dose-dependent manner by approximately 28% at 10 µM and 50% at 100 µM (*p* < 0.05, Figure 6C), whereas paeoniflorin did not alter FABP4 levels significantly at these concentrations (Figure 6D). RU486 blocked GR, leading to reduced FABP4 expression by 46% (*p* = 0.006, Figure 6E), and blocked adipocyte differentiation.

### 3.7. Isoorientin Directly Binds the GR in a Radioligand Binding Assay

**Figure 7:**
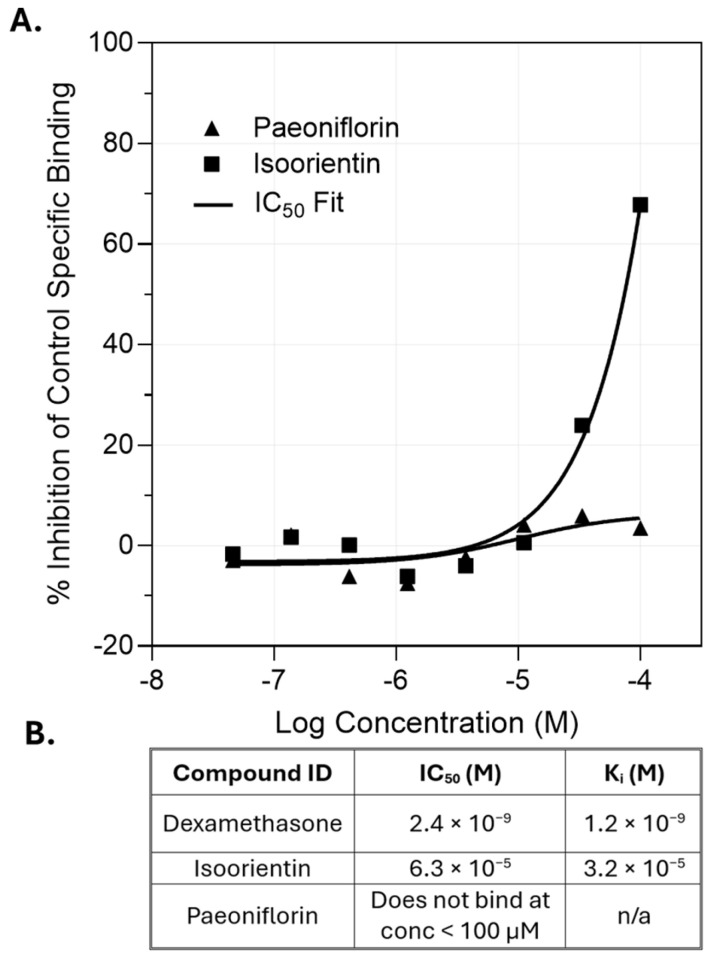
Isoorientin binds GR in a competitive radioligand binding assay. (**A**) Non-linear regression fit curve of paeoniflorin and isoorientin competing off [^3^H] Dex in a radioligand assay. Points represent mean of two biological replicates. (**B**) IC_50_ and K_i_ values of the control dexamethasone and isoorientin. Paeoniflorin showed no measurable binding in the tested range, so a Ki value could not be assigned for it.

To test whether the compounds’ activity occurred through direct GR binding, a competitive binding assay was conducted to determine their IC_50_. Isoorientin displayed measurable binding to (h) GR with an IC_50_ of 6.3 × 10^−5^ M and a K_i_ of 3.2 × 10^−5^ M in the [^3^H] Dex binding assay (Figure 7A). At the highest concentration (1.0 × 10^−4^ M), isoorientin inhibited 67.8% of the control’s specific binding (Figure 7B), exceeding the 50% threshold used to define significant effects.

In contrast, paeoniflorin did not achieve ≥25% inhibition of the control’s specific binding at the highest validated concentration (1.0 × 10^−4^ M) (Figure 7B), and its IC_50_ value was therefore not calculable under these assay conditions.

### 3.8. Computational Ligand Docking of Isoorientin in GR

**Figure 8:**
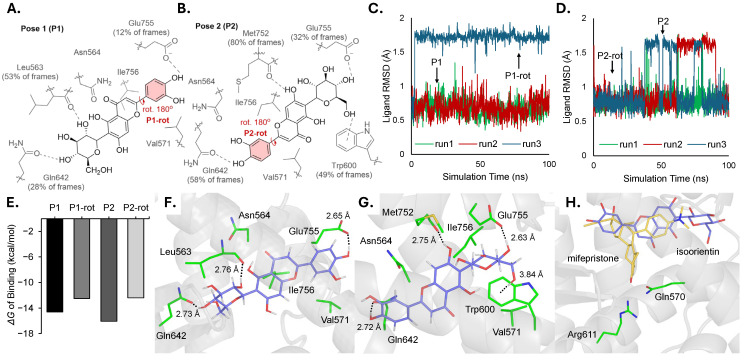
Computational ligand docking shows interaction of isoorientin with GR. (**A**) Ligand rotamer states and residue interaction frequency observed in MD simulations of isoorientin from starting pose 1. (**B**) Ligand rotamer states and residue interaction frequency observed in MD simulations of isoorientin from starting pose 2. (**C**) Identification of different ligand rotamer states from ligand RMSD of Pose 1 (**P1**, **P1-rot**) simulations. (**D**) Identification of different ligand rotamer states from ligand RMSD of Pose 2 (**P2**, **P2-rot**) simulations. (**E**) Binding energies of pose and ligand rotamer states determined by MMGBSA with normal mode entropy correction. (**F**) Pose 1 lowest energy ligand rotamer state (**P1**). (**G**) Pose 2 lowest energy ligand rotamer state (**P2**). (**H**) Docked structures of isoorientin overlayed with a crystal structure of GR-LBD, and RU486 (mifepristone) from Schoch et al. [34]. All distances reported are the heteroatom to heteroatom distance, except interactions with Trp600 which are measured from heteroatom to the centroid of the six membered ring of tryptophan.

In order to understand the mode of binding and inhibition of isoorientin to GR, we carried out a series of computational docking and molecular dynamics studies. The two most populated clusters from Autodock of isoorientin and GR-LBD were used as the starting poses for classical MD simulation. In pose 1 (Figure 8A), the dihydroxybenzyl group is positioned towards the exterior of the binding pocket, while in pose 2 (Figure 8B) the position is flipped, placing the dihydroxybenzyl group towards the interior of the binding pocket.

In pose 1 simulations, two different ligand conformational states are populated, one with an average ligand RMSD of 0.68 Å (**P1**) and another with an average RMSD 1.7 Å (**P1-rot**) (Figure 8C). The two different states observed during MD simulation are generated by a 180° rotation about the C–C bond connecting the fused lactone to the dihydroxybenzyl group of isoorientin. The MMGBSA calculated binding energies were −14.6 kcal/mol and −12.5 kcal/mol for **P1** and **P1-rot**, respectively (Figure 8E, Supplementary Material Table S3).

The most prominent hydrogen bonding interaction for both states was between a backbone oxygen of Leu563 and C2 hydroxyl of the glycosyl group, occurring in 53% of frames with an average distance of 2.76 Å. Additionally, isoorientin displays hydrogen bonding between the glycosyl group and Gln642, with four different hydroxyl groups registering as a hydrogen bond in 9% of frames or more in pose 1 (Figure 8F). The most frequent hydrogen bond was with the C4 hydroxyl, occurring in 28% of frames with an average distance of 2.73 Å. Additionally, the 4-hydroxyl of the dihydroxybenzyl group formed hydrogen bonds with Glu755 in 12% of frames with an average distance of 2.65 Å. However, due to Glu755 being in a more solvent-exposed area of the binding pocket, contacts between the dihydroxyl benzyl group and the solvent were considerably more frequent (74% of frames for the 4-hydroxyl group and 56% of frames for the 3-hydroxyl group). Free energy calculations indicate that isoorientin in pose 1 also experiences additional stabilization, primarily from van der Waals interactions with hydrophobic residues Val571 and Ile756, contributing −1.7 kcal/mol and −2.2 kcal/mol respectively (Supplementary Materials Table S4).

Similarly, the simulations of pose 2 yielded the two different ligand rotamer states generated from the same rotation of the dihydroxybenzyl group as pose 1. The binding energy of pose 2 rotamer state 1 (**P2,** RMSD of 1.6 Å) was calculated to be −16.1 kcal/mol, and the binding energy of pose 2 rotamer state 2 (**P2-rot**, RMSD of 0.8 Å, Figure 8D) was −12.4 kcal/mol (Figure 8E). Pose 2 generally exhibited more persistent hydrogen bonding during MD simulation with GR-LDB than pose 1 (Figure 8G). The most frequent hydrogen bond was between the backbone oxygen of Met752 and the hydroxyl group of the fused lactone of the ligand, occurring in 80% of frames with an average distance of 2.8 Å. Additionally, the hydrogen bonding between Gln642 and ligand was more persistent when the dihydroxybenzyl group was placed deeper into the pocket, interacting with 4-hydroxyl and 3-hydroxyl groups for 58% of frames and 37% of frames, respectively, with both interactions at an average distance of 2.7 Å. The ion-dipole interactions with the side-chain oxygens of Glu755 were also more frequent than in pose 1, with an interaction with the C4 hydroxyl of the glycosyl group observed in 32% of MD frames, at an average distance of 2.6 Å. Pose 2 also demonstrated an interaction with Trp600 with an average oxygen-aryl distance of 3.8 Å across all MD frames, contributing −2.3 kcal/mol stabilization to the overall binding energy (Supplementary Materials Table S5). This contact is characterized by an O–H/π interaction with an average distance of 3.8 Å between the hydroxyl oxygen and the center of mass of the tryptophan six-membered ring. Additionally, hydrophobic interactions with Val571 and Ile756 contributed −1.9 and 1.8 kcal/mol per residue stabilization to the overall binding energy.

The relative energetic similarity between pose 1 and pose 2 indicates that the probability of isoorientin binding to the pocket is similar in both poses. Pose 2 demonstrated a greater frequency of its hydrogen bonds and noncovalent interactions (Figure 8B).

The ligand binding mode of isoorientin does bare some similarities to the binding modes of known antagonists of the GR, mifepristone and dexamethasone. Hydrophobic interactions Leu563, Trp600, and Ile756 as well as hydrogen bonding activity of Asn564 and Gln642 in the binding mode of isoorientin are shared with the two known antagonists [34,49]. However, both known ligands feature hydrogen bonding with Arg611 and Gln570 found deeper in the GR binding pocket. Interactions with these deeper pocket residues were not observed with isoorientin in any of the binding poses considered, indicating the binding of isoorientin is less buried in the pocket than dexamethasone and mifepristone. Additionally, comparing the hydrogen bond occupancy of Gln642 with isoorientin to previous molecular dynamics studies on dexamethasone done using the same crystal structure of the GR [50], it was found that the occupancy of this hydrogen bond with isoorientin was much lower than that of dexamethasone (28% or 58% depending on pose vs. 76% with dexamethasone). Lastly, for isoorientin the binding energy difference resulting from flipping the position of the glycosyl and dihydroxybenzyl in the pocket resulted in a relatively small difference in the binding energy of 1.5 kcal/mol, indicating less specificity in binding. Taken together, the lack of interaction with deep lying residues, lower hydrogen bond occupancy, and small energy differences between significantly different orientations of isoorientin in the binding pocket all support the experimental findings of weaker bonding of isoorientin relative to dexamethasone (Figure 8H).

## 4. Discussion

Women frequently use botanical preparations including white peony and chasteberry, either alone or in combination with other botanicals, in formulations for the management of various gynecological conditions like premenstrual symptoms, perimenopausal complaints and PCOS. Clinical as well as observational data support their beneficial effects in several subsets of patients [16,17,19,22,23]. However, despite their long-standing inclusion in traditional prescriptions and their increasing modern use, the molecular mechanisms by which these botanicals influence steroid hormone signaling pathways remain incompletely elucidated, particularly when crosstalk between receptors is present.

This study demonstrates that paeoniflorin and isoorientin function as antagonists of GR signaling in specific cell models. Both compounds inhibited Dex-induced GR-driven reporter activity without demonstrating agonist activity on a canonical reporter construct, though they differed fundamentally in their binding mechanisms and selective effects on PR signaling.

The literature on steroid receptor signaling in cancer cell lines documents substantial cell-line-specific variations in coactivator expression and preference [49]. For instance, PR and GR preferentially recruit distinct steroid receptor coactivators (SRCs); PR favors SRC-1, while GR preferentially recruits SRC-2/TIF2 [51]. This selectivity determines downstream histone acetylation patterns and chromatin remodeling capacity. Moreover, recent genomic analyses of endometrial versus breast cancer cells reveal profound differences in coactivator cofactor expression patterns and in the genomic distribution of hormone response elements. At the cofactor level, SRC-1 and p300/CBP cofactors show reduced expression in endometrial carcinoma [52]. Moreover T47D A1-2 as a breast cancer cell line expresses PR-A and PR-B; these two isoforms occupy fundamentally different genomic locations despite binding identical DNA motifs, controlling which genomic regions are accessed [53]. The failure of paeoniflorin and isoorientin to inhibit PR signaling in Ishikawa PR-B despite PR antagonism in T47D A1-2 cells expressing GR may be due to the coactivator and cofactor machinery in these two cellular contexts, likely due to interaction with GR. This is noteworthy given that numerous phytochemicals exhibit promiscuous receptor binding profiles.

Co-treatment with either paeoniflorin or isoorientin partially reduced the abundance of Dex-induced transcripts *GILZ* and *DUSP1/MKP1*, confirming that the functional antagonism observed in the reporter assays extends to native GR-responsive genes. When considered alongside the radioligand binding data—showing direct GR binding by isoorientin and not paeoniflorin—these results support a mechanistic distinction in which isoorientin primarily acts as a competitive ligand-binding antagonist, whereas paeoniflorin may operate through indirect or allosteric mechanisms that impair GR transcriptional competence without preventing ligand binding or receptor turnover.

In the receptor binding assay, isoorientin exhibited measurable competitive binding to GR with an IC_50_ of 6.3 × 10^−5^ M, whereas paeoniflorin failed to achieve significant binding at concentrations up to 1.0 × 10^−4^ M. This finding is consistent with emerging literature on selective GR modulators that demonstrate ligand affinity is not sufficient to predict the magnitude or pattern of GR antagonism [54,55]. The field of selective GR modulators (SGRMs) has increasingly recognized that compounds can achieve antagonistic effects through mechanisms including coactivator interference, altered helix-12 positioning, or modulation of cofactor recruitment rather than simple competitive antagonism [56,57,58]. The structural analysis of isoorientin docking revealed that the ligand’s glycosyl group and dihydroxybenzyl moiety engage multiple hydrogen bonding networks with GR-LBD residues, including Leu563, Gln642, and Glu755, with energetically favorable binding poses exhibiting favorable van der Waals interactions with Val571 and Ile756. Critically, the extended positions of isoorientin outside the binding pocket suggest a mechanism where the ligand sterically prevents coactivator binding—a phenomenon analogous to how RU486 (mifepristone) disrupts the receptor conformation required for p300/CBP recruitment [59]. This steric hindrance model aligns with mechanistic studies showing that bulky antagonists can displace helix-12 and occlude the coactivator-binding interface [59,60]. Nevertheless, MD simulations indicate that isoorientin adopts multiple energetically similar but relatively shallow binding orientations, with lower hydrogen bond persistence and fewer interactions with deep GR residues, collectively supporting a weaker binding mode than classical GR antagonists like RU486.

For paeoniflorin, which demonstrated GR antagonism without direct binding, an allosteric or indirect mechanism could be more plausible. Prior pharmacological work on paeoniflorin reported competitive binding to GR with an inhibitor constant around 3.0 × 10^−9^ M in cytosolic assays [61], implying that conditions present in live cells (including cofactor availability, lipid membrane, and post-translational modifications) may enhance its functional potency beyond that which the purified binding assay detected. This is consistent with literature findings on paeoniflorin engaging non-canonical GR pathways through MAPK modulation and GR isoform regulation, which also impairs Dex-induced *GILZ* and *DUSP/MKP1* expression despite the absence of direct receptor binding [62]. Nonetheless, these observations remain descriptive, and other mechanistic pathways—not yet fully characterized—may also contribute to paeoniflorin’s GR-modulatory profile.

Functional consequences of GR antagonism manifest in adipogenic models and link receptor modulation to metabolic outcomes. Metabolic disturbances and hormonal dysregulation are tightly interconnected, with adipose tissue now being recognized as an active endocrine organ that both responds to and shapes systemic sex steroid and glucocorticoid signaling [63]. Dysfunctional adiposity can alter local steroid metabolism, thereby modifying tissue levels of estrogen and progesterone, contributing to conditions such as anovulation, abnormal endometrial function and subfertility. Reciprocally, disturbances in ovarian steroid production or signaling, including altered crosstalk with glucocorticoid pathways, influence lipid storage and fat distribution, creating a bidirectional link between metabolic imbalance and reproductive dysfunction, and using botanicals with complex, multifaceted effects without fully understanding the underlying mechanisms may further contribute to this imbalance. Isoorientin downregulated FABP4 protein (50% at 100 µM), a key fatty acid-binding protein and PPARγ target essential for lipid storage, in differentiated adipocytes compared with paeoniflorin which had no significant effects at a similar concentration, despite comparable suppression of bulk lipid accumulation. The downregulation of FABP4 by isoorientin raises the possibility that direct GR binding may confer additional regulatory advantages beyond simple antagonism, namely, the ability of engaging alternative coactivator or corepressor complexes that specifically modulate FABP4 transcription. Alternatively, the direct GR binding by isoorientin may position the ligand–GR complex to preferentially suppress FABP4 induction relative to other GR targets, a pattern consistent with the selective GR modulator literature where ligand identity determines the set of genes whose regulation is affected [64].

## 5. Conclusions

In conclusion, these findings collectively demonstrate that isoorientin and paeoniflorin are functional GR modulators with distinct features that shape their downstream signaling outcomes. Both compounds attenuated GR-mediated transcriptional activation and target gene expression, with isoorientin doing so in a dose-dependent manner and acting as a weak competitive ligand-binding antagonist, whereas paeoniflorin did not directly compete with the ligand at the GR binding site and will require further studies to elucidate its mechanism of action. From a mechanistic standpoint, both compounds also showed anti-adipogenic activity in 3T3-L1 preadipocytes by reducing lipid accumulation and the late adipogenic marker FABP4, consistent with modulation of GR-associated pathways, but could also involve additional GR-independent mechanisms.

Future work to establish their in vivo pharmacodynamics, receptor-selective modulation and interaction with the GR cofactor network will be essential to clarify their therapeutic potential and contextualize traditional use with modern endocrine therapeutics.

## 6. Limitations

Several limitations should be acknowledged. First, all experiments were conducted in a limited number of cell lines (OVCAR5, Ishikawa PR-B, T47D, and T47D A1-2) and in murine 3T3-L1 adipocytes, which may not necessarily fully reflect GR/PR crosstalk or adipogenic responses in normal human tissues. Second, mechanistic insights are largely based on a canonical HRE-Luc reporter, a small number of GR target genes (*GILZ*, *DUSP1/MKP1*) and FABP4, and thus do not capture broader transcriptomic or non-classical GR/PR signaling effects. Third, the in vitro concentrations of PFL and ISO used may exceed nutritionally achievable exposures from white peony and chasteberry preparations, and possible off-target effects on other nuclear receptors not mentioned here or kinase pathways were not assessed. Finally, the distinction between direct GR binding by ISO and putative indirect or allosteric mechanisms of paeoniflorin remains inferential.

## Data Availability

The raw data supporting the conclusions of this article will be made available by the authors on request.

## Supplementary Materials

The following supporting information can be downloaded at: https://www.mdpi.com/article/10.3390/nu18101491/s1, Figure S1. Hormone receptor expression in the cell line models used in this paper. Figure S2. Isoorientin inhibits GR in OVCAR5 in a dose dependent manner. Figure S3. RU486 downregulate GR-regulated transcripts in OVCAR5 cells via qPCR. Figure S4. Paeoniflorin and isoorientin affect the morphology of 3T3-L1 cells post-differentiation and PFA fixation. Table S1. Primers and sequences of primers used in qPCR analysis. Table S2. Names, catalog numbers, concentration used and sources for the primary antibodies used in protein detection via Western Blot. Table S3. Comparison of binding energies of isoorientin to GR with and without normal mode entropy correction. Table S4. Per residue energy decomposition summaries of the most populated ligand rotamer state of isoorientin Pose 1 State 1. Table S5. Per residue energy decomposition summaries of the most populated ligand rotamer state of isoorientin Pose 2 State 1.

## Abbreviations

The following abbreviations are used in this manuscript:

C/EBPβ	CCAAT/Enhancer-Binding Protein Beta
Dex	Dexamethasone
DHEA	Dehydroepiandrosterone
DM	Differentiation Media
DMSO	Dimethyl Sulfoxide
DTT	Dithiothreitol
*DUSP1/MKP1*	Dual-specificity phosphatase 1/Mitogen-activated protein kinase phosphatase 1
E_2_	17β-estradiol
FABP4	Fatty acid-binding protein 4
FBS	Fetal Bovine serum
GAPDH	Glyceraldehyde-3-phosphate Dehydrogenase
GCs	Glucocorticoids
*GILZ*	Glucocorticoid-induced leucine zipper
GME	Glycylglycine, Magnesium sulphate and EGTA buffer
GnRH	Gonadotropin Releasing Hormone
GR	Glucocorticoid Receptor
HPA	Hypothalamic–Pituitary–Adrenal Axis
HPLC	High-Performance Liquid Chromatography
HRE	Hormone Response Element
IBMX	3-isobutyl-1-methylxanthine
LBD	Ligand Binding Domain
LH	Luteinizing Hormone
MD	Molecular Dynamics
NCoR	Nuclear Corepressor
ORO	Oil Red O
PFA	Paraformaldehyde
P_4_	Progesterone
PBS	Phosphate-Buffered Saline
PCOS	Polycystic Ovary Syndrome
PMS	Premenstrual Syndrome
PPARγ	Peroxisome Proliferator-Activated Receptor Gamma
PR	Progesterone Receptor
PRE	Progesterone Response Element
RIPA	Radioimmunoprecipitation Assay buffer
RMSD	Root-Mean-Square Deviation
RT	Room Temperature
RT-qPCR	Reverse Transcription-quantitative Polymerase Chain Reaction
SDS	Sodium Dodecyl Sulphate
SEM	Standard Error of Mean
SRCs	Steroid Receptor Coactivators
TCM	Traditional Chinese Medicine
