# Supplementary material for: Characterization of Isoorientin and Paeoniflorin as Botanical Glucocorticoid Receptor Modulators from White Peony and Chasteberry
Source: Nutrients. 2026 May 7;18(10):1491. doi: 10.3390/nu18101491 (PMC13209654; doi:10.3390/nu18101491)
Supplement: Supplementary file 1 [file nutrients-18-01491-s001.zip › nutrients-4262390-supplementary.pdf]

Supplementary information

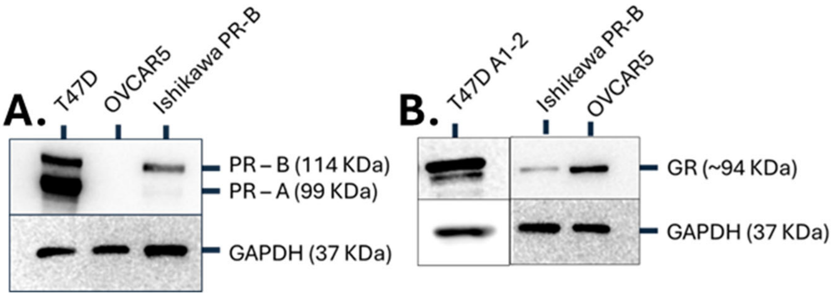

Figure S1: Hormone receptor expression in the cell line models used in this paper. (A) A western blot of T47D, OVCAR5 and Ishikawa PR-B cell lysates detecting the endogenous expression of progesterone receptor (PR) isoforms; PR-A at ~99 KDa and PR-B at ~114 KDa, using GAPDH as a loading control. (B) A western blot of T47D A1-2, Ishikawa PR-B and OVCAR5 cell lysates detecting endogenous glucocorticoid receptor (GR) expression at ~94 KDa, using GAPDH as a loading control.

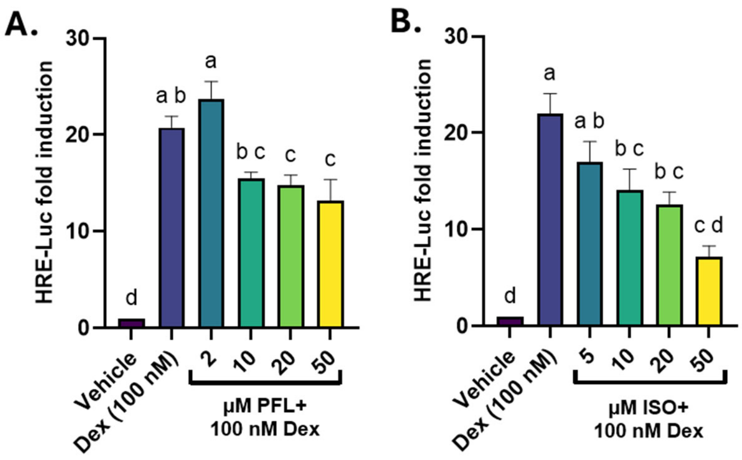

Figure S2: Isoorientin inhibits GR in OVCAR5 in a dose dependent manner (A) HRE-Luc assay in OVCAR5 cells treated with vehicle (0.1% DMSO), 100 nM Dex, 2, 10, 20, 50 µM PFL in the presence of 100 nM Dex for 24 hrs. (B) HRE-Luc assay in OVCAR5 cells treated with vehicle, 100 nM Dex, 5,10, 20, 50 µM ISO in the presence of 100 nM Dex. Dex, PFL, and ISO are dissolved in DMSO at a final concentration of 0.1%. N≥3. Data represent mean ± SEM. Letters indicate results of one-way ANOVA with Tukey's post hoc analysis; groups not sharing a letter differ at $P < 0.05$.

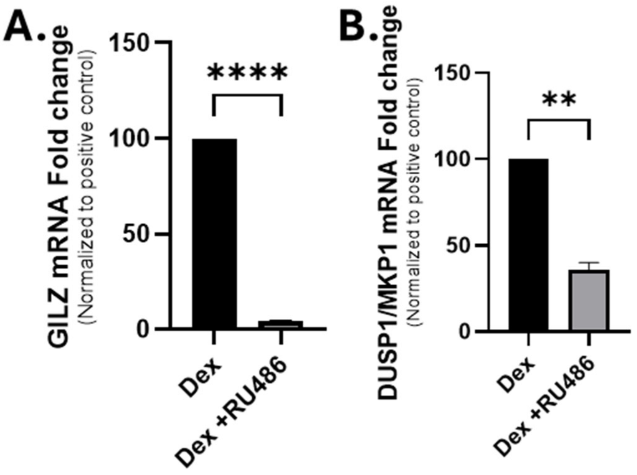

Figure S3: RU486 downregulate GR-regulated transcripts in OVCAR5 cells via qPCR. (A) GILZ and (B) DUSP1/MKP1 in cells treated with 30 nM Dex combined with 1 μM RU486 for 6 hrs. N=3. The values represent the mean ± SEM. Significant values were determined by a student t-test, * $P<0.05$, ** $P<0.01$ relative to control.

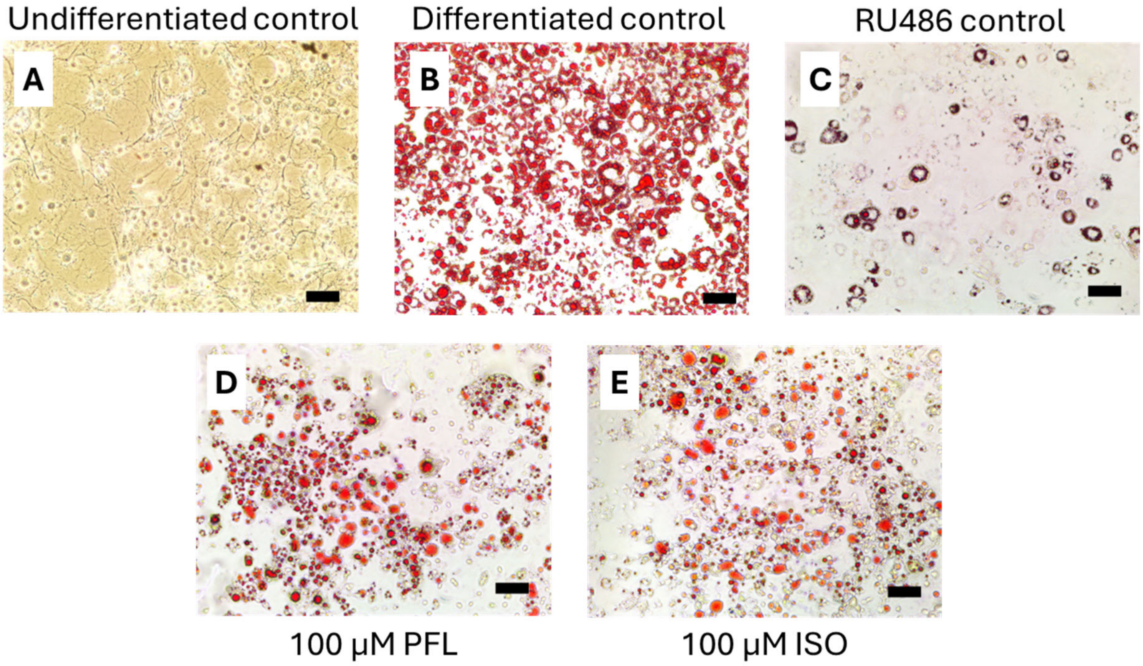

Figure S4: Paeoniflorin and isoorientin affect the morphology of 3T3-L1 cells post-differentiation and PFA fixation. The cells are visibly smaller and pack less lipids within. Brightfield images of cells stained with Oil-Red O (ORO) stain on treatment conditions of (A) 0.1% DMSO leading to undifferentiated cells, or (B) DMI cocktail leading to differentiated control, or (C) 10 µM RU486, a GR inhibitor, or (D) 100 µM PFL, or (E) 100 µM ISO. The images were taken at a magnification of 10X.

Table S1: Primers and sequences of primers used in qPCR analysis.

Gene	Sequence
GILZ	Forward 5'-TCCGTTAAGCTGGACAACAGT-3'<br>Reverse 5'-ATGGCCTGTTCGATCTTGTT-3'
MKP1/DUSP1	Forward 5' -CCTGACAGCGCGGAATCT-3'<br>Reverse 5'- GATTTCCACCGGGCCAC-3'
GAPDH	Forward 5' -ATGGGGAAGGTGAAGGTCG-3'<br>Reverse 5' -GGGGTCATTGATGGCAACAATA-3'

Table S2: Names, catalog numbers, concentration used and sources for the primary antibodies used in protein detection via Western Blot.

Antibody name	Catalog no	Concentration	Source
FABP4	2120	1:1000	Cell Signaling Technology, Danvers, USA
GR	12041S	1:1000	Cell Signaling Technology, Danvers, USA
PR	A0321	1:1000	Abclonal, China
GAPDH	2118	1:10000	Cell Signaling Technology, Danvers, USA
Vinculin	4650S	1:1000	Cell Signaling Technology, Danvers, USA

Table S3: Comparison of binding energies of isoorientin to GR with and without normal mode entropy correction.

Pose and Rotamer State	ΔG of binding w/ nmode (kcal/mol)	ΔG of binding w/o nmode (kcal/mol)
Pose 1 Rotamer State 1	-14.6274 +/- 6.3730	-37.2366+/- 4.1228
Pose 1 Rotamer State 2	-12.4925 +/- 9.5281	-35.8272 +/- 4.1224
Pose 2 Rotamer State 1	-12.3945 +/- 7.2023	-36.7050+/- 4.9057
Pose 2 Rotamer State 2	-16.0979 +/- 6.5287	-38.5193 +/- 4.419

The values for the ΔG of binding with the normal mode entropy correction (used in main text discussion) were found to be 20–25 kcal/mol higher than the values for the ΔG of binding computed without the entropy correction. However, the energetic ranking of the different poses and ligand rotamer states was conserved across both methods.

Table S4: Per residue energy decomposition summaries of the most populated ligand rotamer state of isoorientin Pose 1 State 1:

Residue	Van der Waals	Electrostatic	Polar Solv	Non-polar Solv	Total
**Leu563**	-1.109 +/- 0.609	-2.222 +/-1.796	1.309 +/-0.549	-0.158 +/- 0.034	-2.180 +/- 1.243
Asn564	-2.277 +/- 0.383	-0.279 +/- 0.781	1.625 +/- 0.710	-0.156 +/- 0.037	-1.087 +/- 0.467
**Val571**	-1.286 +/- 0.279	0.099 +/- 0.167	-0.305 +/- 0.133	-0.206 +/- 0.044	-1.698 +/- 0.355
Trp600	-1.406 +/- 0.399	-0.373 +/-0.255	0.611 +/- 0.219	-0.160 +/- 0.034	-1.328 +/- 0.353
Met601	-1.576 +/- 0.399	-0.145 +/-0.286	0.396 +/- 0.201	0.101 +/- 0.029	-1.426 +/- 0.416
**Gln642**	-0.487 +/- 0.818	-4.478 +/2.542	3.216 +/- 0.963	-0.163 +/- 0.050	-1.912 +/- 1.223
Met752	-1.509 +/- 0.346	-0.694 +/- 0.487	1.500 +/- 0.439	-0.156 +/- 0.036	-0.860 +/- 0.455
**Glu755**	-1.471 +/- 0.614	-3.236 +/- 4.321	3.332 +/- 3.492	-0.315 +/- 0.046	-1.690 +/- 0.706
**Ile756**	-1.903 +/- 0.374	-0.343 +/- 0.125	0.238 +/- 0.113	-0.224 +/- 0.035	-2.232 +/- 0.392

Table S5: Per residue energy decomposition summaries of the most populated ligand rotamer state of isoorientin Pose 2 State 1:

Residue	Van der Waals	Electrostatic	Polar Solv	Non-polar Solv	Total
Leu563	-1.998 +/- 0.368	0.324 +/- 0.389	0.459 +/- 0.452	-0.209 +/- 0.047	-1.424 +/- 0.395
Asn564	-2.277 +/- 0.383	-0.279 +/- 0.781	1.625 +/- 0.710	-0.156 +/- 0.037	-1.087 +/- 0.467
**Val571**	-1.442 +/- 0.285	-0.387 +/- 0.218	0.141 +/- 0.195	-0.217 +/- 0.041	-1.905 +/- 0.331
**Trp600**	-2.688 +/- 0.427	-0.018 +/- 0.618	0.637 +/- 0.390	-0.232 +/- 0.041	-2.301 +/- 0.573
Met601	-0.366 +/- 0.166	-0.008 +/- 0.160	0.118 +/- 0.152	-0.012 +/- 0.024	-0.269 +/- 0.147
**Gln642**	-0.547 +/- 1.001	-2.830 +/- 3.376	2.616 +/- 1.358	-0.214 +/- 0.066	-0.975 +/- 1.368
**Met752**	-0.931 +/- 0.558	-3.417 +/- 0.995	1.815 +/- 0.567	-0.143 +/- 0.045	-2.675 +/- 0.696
**Glu755**	-1.323 +/- 0.853	-5.680 +/- 7.080	5.172 +/- 5.368	-0.316 +/- 0.049	-2.148 +/- 1.323
**Ile756**	-1.791 +/- 0.357	0.467 +/- 0.267	-0.345 +/- 0.184	-0.185 +/- 0.042	-1.854 +/- 0.371
